# Evaluation and improvement of the National Early Warning Score (NEWS2) for COVID-19: a multi-hospital study

**DOI:** 10.1186/s12916-020-01893-3

**Published:** 2021-01-21

**Authors:** Ewan Carr, Rebecca Bendayan, Daniel Bean, Matt Stammers, Wenjuan Wang, Huayu Zhang, Thomas Searle, Zeljko Kraljevic, Anthony Shek, Hang T. T. Phan, Walter Muruet, Rishi K. Gupta, Anthony J. Shinton, Mike Wyatt, Ting Shi, Xin Zhang, Andrew Pickles, Daniel Stahl, Rosita Zakeri, Mahdad Noursadeghi, Kevin O’Gallagher, Matt Rogers, Amos Folarin, Andreas Karwath, Kristin E. Wickstrøm, Alvaro Köhn-Luque, Luke Slater, Victor Roth Cardoso, Christopher Bourdeaux, Aleksander Rygh Holten, Simon Ball, Chris McWilliams, Lukasz Roguski, Florina Borca, James Batchelor, Erik Koldberg Amundsen, Xiaodong Wu, Georgios V. Gkoutos, Jiaxing Sun, Ashwin Pinto, Bruce Guthrie, Cormac Breen, Abdel Douiri, Honghan Wu, Vasa Curcin, James T. Teo, Ajay M. Shah, Richard J. B. Dobson

**Affiliations:** 1 Department of Biostatistics and Health Informatics, Institute of Psychiatry, Psychology and Neuroscience (IoPPN), King’s College London, 16 De Crespigny Park, London, SE5 8AF UK; 2 NIHR Biomedical Research Centre at South London and Maudsley NHS Foundation Trust and King’s College London, London, UK; 3 Health Data Research UK London, University College London, London, UK; 4 Clinical Informatics Research Unit, University of Southampton, Coxford Rd., Southampton, SO16 5AF UK; 5 NIHR Biomedical Research Centre at University Hospital Southampton NHS Trust, Coxford Road, Southampton, UK; 6 UHS Digital, University Hospital Southampton, Tremona Road, Southampton, SO16 6YD UK; 7 School of Population Health and Environmental Sciences, King’s College London, London, UK; 8 Usher Institute, University of Edinburgh, Edinburgh, UK; 9 Department of Clinical Neuroscience, Institute of Psychiatry, Psychology and Neuroscience, King’s College London, London, UK; 10 UCL Institute for Global Health, University College London Hospitals NHS Trust, London, UK; 11 University Hospitals Bristol and Weston NHS Foundation Trust, Bristol, UK; 12 Department of Pulmonary and Critical Care Medicine, People’s Liberation Army Joint Logistic Support Force 920th Hospital, Kunming, Yunnan China; 13 King’s College Hospital NHS Foundation Trust, London, UK; 14 School of Cardiovascular Medicine & Sciences, King’s College London British Heart Foundation Centre of Excellence, London, SE5 9NU UK; 15 UCL Division of Infection and Immunity, University College London Hospitals NHS Trust, London, UK; 16 Institute of Health Informatics, University College London, London, UK; 17 NIHR Biomedical Research Centre at University College London Hospitals NHS Foundation Trust, London, UK; 18 College of Medical and Dental Sciences, Institute of Cancer and Genomics, University of Birmingham, Birmingham, UK; 19 Institute of Translational Medicine, University Hospitals Birmingham NHS Foundation Trust, Birmingham, UK; 20 Health Data Research UK Midlands, Birmingham, UK; 21 Department of Medical Biochemistry, Blood Cell Research Group, Oslo University Hospital, Oslo, Norway; 22 Oslo Centre for Biostatistics and Epidemiology, Faculty of Medicine, University of Oslo, Oslo, Norway; 23 Department of Acute Medicine, Oslo University Hospital and Institute of Clinical Medicine, University of Oslo, Oslo, Norway; 24 University Hospitals Birmingham NHS Foundation Trust, Birmingham, UK; 25 Department of Engineering Mathematics, University of Bristol, Bristol, UK; 26 Department of Pulmonary and Critical Care Medicine, Shanghai East Hospital, Tongji University, Shanghai, China; 27 Department of Pulmonary and Critical Care Medicine, Taikang Tongji Hospital, Wuhan, China

**Keywords:** NEWS2 score, Blood parameters, COVID-19, Prediction model

## Abstract

**Background:**

The National Early Warning Score (NEWS2) is currently recommended in the UK for the risk stratification of COVID-19 patients, but little is known about its ability to detect severe cases. We aimed to evaluate NEWS2 for the prediction of severe COVID-19 outcome and identify and validate a set of blood and physiological parameters routinely collected at hospital admission to improve upon the use of NEWS2 alone for medium-term risk stratification.

**Methods:**

Training cohorts comprised 1276 patients admitted to King’s College Hospital National Health Service (NHS) Foundation Trust with COVID-19 disease from 1 March to 30 April 2020. External validation cohorts included 6237 patients from five UK NHS Trusts (Guy’s and St Thomas’ Hospitals, University Hospitals Southampton, University Hospitals Bristol and Weston NHS Foundation Trust, University College London Hospitals, University Hospitals Birmingham), one hospital in Norway (Oslo University Hospital), and two hospitals in Wuhan, China (Wuhan Sixth Hospital and Taikang Tongji Hospital). The outcome was severe COVID-19 disease (transfer to intensive care unit (ICU) or death) at 14 days after hospital admission. Age, physiological measures, blood biomarkers, sex, ethnicity, and comorbidities (hypertension, diabetes, cardiovascular, respiratory and kidney diseases) measured at hospital admission were considered in the models.

**Results:**

A baseline model of ‘NEWS2 + age’ had poor-to-moderate discrimination for severe COVID-19 infection at 14 days (area under receiver operating characteristic curve (AUC) in training cohort = 0.700, 95% confidence interval (CI) 0.680, 0.722; Brier score = 0.192, 95% CI 0.186, 0.197). A supplemented model adding eight routinely collected blood and physiological parameters (supplemental oxygen flow rate, urea, age, oxygen saturation, C-reactive protein, estimated glomerular filtration rate, neutrophil count, neutrophil/lymphocyte ratio) improved discrimination (AUC = 0.735; 95% CI 0.715, 0.757), and these improvements were replicated across seven UK and non-UK sites. However, there was evidence of miscalibration with the model tending to underestimate risks in most sites.

**Conclusions:**

NEWS2 score had poor-to-moderate discrimination for medium-term COVID-19 outcome which raises questions about its use as a screening tool at hospital admission. Risk stratification was improved by including readily available blood and physiological parameters measured at hospital admission, but there was evidence of miscalibration in external sites. This highlights the need for a better understanding of the use of early warning scores for COVID.

**Supplementary Information:**

The online version contains supplementary material available at 10.1186/s12916-020-01893-3.

## Key messages

- The National Early Warning Score (NEWS2), currently recommended for stratification of severe COVID-19 disease in the UK, showed poor-to-moderate discrimination for medium-term outcomes (14-day transfer to intensive care unit (ICU) or death) amongst COVID-19 patients.
- Risk stratification was improved by the addition of routinely measured blood and physiological parameters routinely at hospital admission (supplemental oxygen, urea, oxygen saturation, C-reactive protein, estimated glomerular filtration rate, neutrophil count, neutrophil/lymphocyte ratio) which provided moderate improvements in a risk stratification model for 14-day ICU/death.
- This improvement over NEWS2 alone was maintained across multiple hospital trusts, but the model tended to be miscalibrated with risks of severe outcomes underestimated in most sites.
- We benefited from existing pipelines for informatics at King’s College Hospital such as CogStack that allowed rapid extraction and processing of electronic health records. This methodological approach provided rapid insights and allowed us to overcome the complications associated with slow data centralisation approaches.

## Background

As of 9 December 2020, there have been > 67 million confirmed cases of COVID-19 disease worldwide [1]. While approximately 80% of infected individuals have mild or no symptoms [2], some develop severe COVID-19 disease requiring hospital admission. Within the subset of those requiring hospitalisation, early identification of those who deteriorate and require transfer to an intensive care unit (ICU) for organ support or may die is vital.

Currently, available risk scores for deterioration of acutely ill patients include (i) widely used generic ward-based risk indices such as the National Early Warning Score (NEWS2, [3]), (ii) the Modified Sequential Organ Failure Assessment (mSOFA) [4] and Quick Sequential Organ Failure Assessment [5] scoring systems, and (iii) the pneumonia-specific risk index, CURB-65 [6] which combines physiological observations with limited blood markers and comorbidities. NEWS2 is a summary score of six physiological parameters or ‘vital signs’ (respiratory rate, oxygen saturation, systolic blood pressure, heart rate, level of consciousness, temperature and supplemental oxygen dependency) used to identify patients at risk of early clinical deterioration in the United Kingdom (UK) National Health Service (NHS) hospitals [7, 8] and primary care. Some components (in particular, patient temperature, oxygen saturation, and supplemental oxygen dependency) have been associated with COVID-19 outcomes [2], but little is known about their predictive value for COVID-19 disease severity in hospitalised patients [9]. Additionally, a number of COVID-19-specific risk indices are being developed [10, 11] as well as unvalidated online calculators [12], but generalisability is unknown [13]. A Chinese study has suggested a modified version of NEWS2 with the addition of age only [14] but without any data on performance. With near-universal usage of NEWS2 in UK NHS Trusts since March 2019 [15], a minor adaptation to NEWS2 would be relatively easy to implement.

As the SARS-Cov2 pandemic has progressed, a number of risk prediction models to support clinical decisions, triage, and care in hospitalised patients have been proposed [13] incorporating potentially useful blood biomarkers [2, 16–19]. These include neutrophilia and lymphopenia, particularly in older adults [11, 18, 20, 21]; neutrophil-to-lymphocyte ratio [22]; C-reactive protein (CRP) [13]; lymphocyte-to-CRP ratio [22]; markers of liver and cardiac injury such as alanine aminotransferase (ALT), aspartate aminotransferase (AST), and cardiac troponin [23]; and elevated d-dimers, ferritin and fibrinogen [2, 6, 8].

Our aim is to evaluate the NEWS2 score and identify which clinical and blood biomarkers routinely measured at hospital admission can improve medium-term risk stratification of severe COVID-19 outcome at 14 days from hospital admission. Our specific objectives were as follows:

1. To explore independent associations of routinely measured physiological and blood parameters (including NEWS2 parameters) at hospital admission with disease severity (ICU admission or death at 14 days from hospital admission), adjusting for demographics and comorbidities
2. To develop a prediction model for severe COVID-19 outcomes at 14 days combining multiple blood and physiological parameters
3. To compare the discrimination, calibration, and clinical utility of the resulting model with NEWS2 score and age alone using (i) internal validation and (ii) external validation at seven UK and international sites

A recent systematic review found that most existing prediction models for COVID-19 had a high risk of bias due to non-representative samples, model overfitting, or poor reporting [13]. The analyses presented here build upon our earlier work [24] which suggested that adding age and common blood biomarkers to the NEWS2 score could improve risk stratification in patients hospitalised with COVID-19. While incorporating external validation, this preliminary work was limited in that the training sample comprised 439 patients (the cohort available at the time of model development). In the present study, we (i) expand the cohort used for model development to all 1276 patients at King’s College Hospital (KCH), (ii) use hospital admission (rather than symptom onset) as the index date, (iii) consider shorter-term outcomes (3-day ICU/death), (iv) improve the reporting of model calibration and clinical utility, and (v) increase the number of external sites from three to seven.

## Methods

### Study cohorts

The KCH training cohort (*n* = 1276) was defined as all adult inpatients testing positive for severe acute respiratory syndrome coronavirus 2 (SARS-Cov2) by reverse transcription polymerase chain reaction (RT-PCR) between 1 March and 31 April 2020 at two acute hospitals (King’s College Hospital and Princess Royal University Hospital) in South East London (UK) of Kings College Hospital NHS Foundation Trust (KCH). All patients included in the study had symptoms consistent with COVID-19 (e.g. cough, fever, dyspnoea, myalgia, delirium, diarrhoea). For external validation purposes, we used seven cohorts:

1. Guy’s and St Thomas’ Hospital NHS Foundation Trust (GSTT) of 988 cases (3 March 2020 to 26 August 2020)
2. University Hospitals Southampton NHS Foundation Trust (UHS) of 633 cases (7 March to 6 June 2020)
3. University Hospitals Bristol and Weston NHS Foundation Trust (UHBW) of 190 cases (12 March to 11 June 2020)
4. University College Hospital London (UCH) of 411 cases (1 February to 30 April 2020)
5. University Hospitals Birmingham (UHB) of 1037 cases (1 March to 31 June 2020)
6. Oslo University Hospital (OUH) of 163 cases (6 March to 13 June 2020)
7. Wuhan Sixth Hospital and Taikang Tongji Hospital of 2815 cases (4 February 2020 to 30 March 2020)

Data were extracted from structured and/or unstructured components of electronic health records (EHR) in each site as detailed below.

### Measures

#### Outcome

For all sites, the outcome was severe COVID-19 disease at 14 days following hospital admission, categorised as transfer to the ICU/death (WHO-COVID-19 Outcomes Scales 6–8) vs. not transferred to the ICU/death (scales 3–5) [25]. For nosocomial patients (patients with symptom onset after hospital admission), the endpoint was defined as 14 days after symptom onset. Dates of hospital admission, symptom onset, ICU transfer, and death were extracted from electronic health records or ascertained manually by a clinician.

#### Blood and physiological parameters

We included blood and physiological parameters that were routinely obtained at hospital admission and which are routinely available in a wide range of national and international hospital and community settings. Measures available for fewer than 30% of patients were not considered (including Troponin-T, Ferritin, d-dimers and glycated haemoglobin (HbA1c), Glasgow Coma Scale score). We excluded creatinine since this parameter correlates highly (*r* > 0.8) with, and is used in the derivation of, estimated glomerular filtration rate. We excluded white blood cell count (WBCs) which is highly correlated with neutrophil and lymphocyte counts.

The candidate blood parameters therefore comprised albumin (g/L), C-reactive protein (CRP; mg/L), estimated glomerular filtration rate (GFR; mL/min), haemoglobin (g/L), lymphocyte count (× 10^9^/L), neutrophil count (× 10^9^/L), platelet count (PLT; × 10^9^/L), neutrophil-to-lymphocyte ratio (NLR), lymphocyte-to-CRP ratio [22], and urea (mmol/L). The candidate physiological parameters included the NEWS2 total score, as well as the following parameters: respiratory rate (breaths per minute), oxygen saturation (%), supplemental oxygen flow rate (L/min), diastolic blood pressure (mmHg), systolic blood pressure (mmHg), heart rate (beats/min), and temperature (°C). For all parameters, we used the first available measure up to 48 h following hospital admission.

#### Demographics and comorbidities

Age, sex, ethnicity and comorbidities were considered. Self-defined ethnicity was categorised as White vs. non-White (Black, Asian, or other minority ethnic) and patients with ethnicity recorded as ‘unknown/mixed/other’ were excluded (*n* = 316; 25%). Binary variables were derived for comorbidities: hypertension, diabetes, heart disease (heart failure and ischemic heart disease), respiratory disease (asthma and chronic obstructive pulmonary disease (COPD)), and chronic kidney disease.

### Data processing

#### King’s College Hospital

Data were extracted from the structured and unstructured components of the electronic health record (EHR) using natural language processing (NLP) tools belonging to the CogStack ecosystem [26], namely MedCAT [27] and MedCATTrainer [28]. The CogStack NLP pipeline captures negation, synonyms, and acronyms for medical Systematised Nomenclature of Medicine Clinical Terms (SNOMED-CT) concepts as well as surrounding linguistic context using deep learning and long short-term memory networks. MedCAT produces unsupervised annotations for all SNOMED-CT concepts (Additional file 1: Table S1) under parent terms Clinical Finding, Disorder, Organism, and Event with disambiguation, pre-trained on MIMIC-III [29]. Starting from our previous model [30], further supervised training improved detection of annotations and meta-annotations such as experiencer (is the annotated concept experienced by the patient or other), negation (is the concept annotated negated or not), and temporality (is the concept annotated in the past or present) with MedCATTrainer. Meta-annotations for hypothetical, historical, and experiencer were merged into “Irrelevant” allowing us to exclude any mentions of a concept that did not directly relate to the patient currently. Performance of the NLP pipeline for comorbidities mentioned in the text was evaluated on 4343 annotations in 146 clinical documents by a clinician (JT). F1 scores, precision, and recall are presented in Additional file 2: Table S2.

#### Guy’s and St Thomas’ NHS Foundation Trust

Electronic health records from all patients admitted to Guy’s and St Thomas’ NHS Foundation Trust who had a positive COVID-19 test result between 3 March and 21 May 2020, inclusive, were identified. Data were extracted using structured queries from six complementary platforms and linked using unique patient identifiers. Data processing was performed using Python 3.7 [31]. The process and outputs were reviewed by a study clinician.

#### University Hospitals Southampton

Data were extracted from the structured components of the UHS CHARTS EHR system and data warehouse. Data were transformed into the required format for validation purposes using Python 3.7 [31]. Diagnosis and comorbidity data of interest were gathered from the International Statistical Classification of Diseases (ICD-10) coded data. No unstructured data extraction was required for validation purposes. The process and outputs were reviewed by an experienced clinician prior to analysis.

#### University Hospitals Bristol and Weston NHS Foundation Trust

Data were extracted from UHBW electronic health records system (Medway). ICD-10 codes were used for diagnosis and comorbidity data. Data were transformed in line with project specifications and exported for analysis in Python 3.7 [31].

#### University College Hospital London

Dates of hospital admission, symptom onset, ICU transfer, and death were extracted from electronic health records. The outcome (14-day ICU/death) was defined in UCLH as ‘initiation of ventilatory support (continuous positive airway pressure, non-invasive ventilation, high-flow nasal cannula oxygen, invasive mechanical ventilation, or extracorporeal membrane oxygenation) or death’ which is consistent WHO-COVID-19 Outcomes Scales 6–8.

#### Wuhan cohort

Demographic, premorbid conditions, clinical symptoms or signs at presentation, laboratory data, and treatment and outcome data were extracted from electronic medical records using a standardised data collection form by a team of experienced respiratory clinicians, with double data checking and involvement of a third reviewer where there was disagreement. Anonymised data was entered into a password-protected computerised database.

#### University Hospitals Birmingham

Dates of hospital admission, symptom onset, ICU transfer, and death were extracted from electronic health records using the Prescribing Information and Communications System (PICS) system. The extracted data was transformed into the required format for validation purposes using Python 3.8 [31]. Diagnosis and comorbidity data of interest were gathered from ICD-10 coded data. The outcomes (3- and 14-day ICU/death) were defined consistent with WHO-COVID-19 Outcomes Scales 6–8.

#### Oslo University Hospital

All admitted patients with confirmed COVID-19 by positive SARS-CoV2 PCR were included in a quality registry. Data input into the register was manual. Register data was supplemented with test results from the laboratory information system (LIS) by matching exported Excel files from the register with exported Excel files from LIS. The fidelity of the match was checked against the original data source manually for a small number of patients. Only patients with symptoms consistent with COVID-19 were included in the study.

### Statistical analyses

All continuous parameters were winsorized (at 1% and 99%) and scaled (mean = 0; standard deviation = 1) to facilitate interpretability and comparability [32]. Logarithmic or square root transformations were applied to skewed parameters. To explore independent associations of blood and physiological parameters with 14-day ICU/death (objective 1), we used logistic regression with Firth’s bias reduction method [33]. Each parameter was tested independently, adjusted for age and sex (model 1), and then additionally adjusted for comorbidities (model 2). *P* values were adjusted using the Benjamini-Hochberg procedure to keep the false discovery rate (FDR) at 5% [34].

To evaluate NEWS2 and identify parameters that could improve prediction of severe COVID-19 outcomes (objectives 2 and 3), we used regularised logistic regression with a least absolute shrinkage and selection operator (LASSO) estimator that shrinks parameters according to their variance, reduces overfitting, and enables automatic variable selection [35]. The optimal degree of regularisation was determined by identifying a tuning parameter *λ* using cross-validation. To avoid overfitting and to reduce the number of false-positive predictors, *λ* was selected to give a model with an area under the receiver operating characteristic curve (AUC) one standard error below the ‘best’ model. To evaluate the predictive performance of our model on new cases of the same underlying population (internal validation), we performed nested cross-validation (10-folds the for inner loop; 10-folds/1000 repeats for the outer loop). Discrimination was assessed using AUC and Brier score. Missing feature information was imputed using *k*-nearest neighbour (kNN) imputation (*k* = 5). All steps (feature selection, winsorizing, scaling, and kNN imputation) were incorporated within the model development and selection process to avoid data leakage that would otherwise result in optimistic performance measures [36]. All analyses were conducted with Python 3.8 [31] using the statsmodels [37] and Scikit-Learn [38] packages.

We evaluated the transportability of the derived regularised logistic regression model in external validation samples from GSTT (*n* = 988), UHS (*n* = 633), UHBW (*n* = 190), UCH (*n* = 411), UHB (*n* = 1037), OUH (*n* = 163), and Wuhan (*n* = 2815). Validation used LASSO logistic regression models trained on the KCH training sample, with code and pre-trained models shared via GitHub.^1^ Models were assessed in terms of discrimination (AUC, sensitivity, specificity, Brier score), calibration, and clinical utility (decision curve analysis, number needed to evaluate) [32, 39]. Moderate calibration was assessed by plotting model-predicted probabilities (*x*-axis) against observed proportions (*y*-axis) with locally estimated scatterplot smoothing (LOESS) and logistic curves [40]. Clinical utility was assessed using decision curve analysis where ‘net benefit’ was plotted against a range of threshold probabilities. Unlike diagnostic performance measures, decision curves incorporate preferences of the clinician and patient. The threshold probability (*p*_*t*_) is where the expected benefit of treatment is equal to the expected benefit of avoiding treatment [41]. Net benefit was calculated by counting the number of true positives (predicted risk > *p*_*t*_ and experienced severe COVID-19 outcome) and false positives (predicted risk > *p*_*t*_ but did not experience severe COVID-19 outcome) and using the below formula:

$$ \mathrm{Net}\ \mathrm{benefit}=\frac{\mathrm{True}\ \mathrm{positives}}{N}-\frac{\mathrm{False}\ \mathrm{positives}}{N}\times \frac{p_t}{1-{p}_t} $$

Our model was developed as a screening tool, to identify at hospital admission patients at risk of more severe outcomes. The intended treatment for patients with a positive result from this model would be further examination by a clinician, who would make recommendations regarding appropriate treatment (e.g. earlier transfer to the ICU, intensive monitoring, treatment). We compared the decision curve from our model to two extreme cases of ‘treat none’ and ‘treat all’. The ‘treat none’ (i.e. routine management) strategy implies that no patients would be selected for further examination by a clinician; the ‘treat all’ strategy (i.e. intensive management) implies that all patients would undergo further assessment. A model is clinically beneficial if the model-implied net benefit is greater than either the ‘treat none’ or ‘treat all’ strategies.

Since the intended strategy involves a further examination by a clinician, and is therefore low risk, our emphasis throughout is on avoiding false negatives (i.e. failing to detect a severe case) at the expense of false positives. We therefore used thresholds of 30% and 20% (for 14-day and 3-day outcomes, respectively) to calculate sensitivity and specificity. This gave a better balance of sensitivity vs. specificity and reflected the clinical preference to avoid false negatives for the proposed screening tool.

#### Sensitivity analyses

We conducted five sensitivity analyses. First, to explore the ability of NEWS2 to predict shorter-term severe COVID-19 outcome, we developed models for ICU transfer/death at 3 days following hospital admission. All steps described above were repeated, including training (feature selection) and external validation. Second, following recent studies suggesting sex differences in COVID-19 outcome [18], we tested interactions between each physiological and blood parameters and sex using likelihood-ratio tests. Third, we repeated all models with adjustment for ethnicity in the subset of individuals with available data for ethnicity (*n* = 960 in the KCH training sample). Fourth, to explore the differences between community-acquired vs. nosocomial infection, we repeated all models after excluding 153 nosocomial patients (*n* = 1123). Finally, we considered an alternative baseline model of ‘NEWS2 only’. Our primary analyses used a baseline model of ‘NEWS2 + age’ because NEWS2 is rarely used in isolation for prognostication and treatment decisions will incorporate other patient characteristics such as age.

## Results

### Descriptive analyses

The KCH training cohort comprised 1276 patients admitted with a confirmed diagnosis of COVID-19 (from 1 March to 31 April 2020) of whom 389 (31%) were transferred to the ICU or died within 14 days of hospital admission, respectively. The validation cohorts comprised 6237 patients across seven sites. At UK NHS trusts, 30 to 42% of patients were transferred to the ICU or died within 14 days of admission. Disease severity was lower in the Wuhan sample, where 4% were transferred to the ICU or died. Table 1 presents the demographic and clinical characteristics of the training and validation cohorts. The UK sites were similar in terms of age and sex, with patients tending to be older (median age 59–74) and male (58 to 63%) but varied in the proportion of patients of non-White ethnicity (from 10% at UHS to 40% at KCH and UCH). Blood and physiological parameters were broadly consistent across UK sites.

**Table 1:** Patient characteristics of the training/validation cohorts

	Training cohort		Validation cohorts (*n* = 6237)													
	KCH (*n* = 1276)		UHS (*n* = 633)		UCH (*n* = 411)		GSTT (*n* = 988)		UHBW (*n* = 190)		Wuhan (*n* = 2815)		UHB (*n* = 1037)		Oslo (*n* = 163)	
**COVID-19 WHO Score 6–8 (ICU/death)**	*N* avail.	*N* (%)	*N* avail.	*N* (%)	*N* avail.	*N* (%)	*N* avail.	*N* (%)	*N* avail.	*N* (%)	*N* avail.	*N* (%)	*N* avail.	*N* (%)	*N* avail.	*N* (%)
3 days	1276	163 (12.8%)	633	109 (17.2%)	411	120 (29.0%)	988	289 (29.3%)	190	32 (16.8%)	2815	58 (2.1%)	1037	169 (16.3%)	163	27 (16.3%)
14 days	1276	389 (30.5%)	633	223 (35.2%)	411	171 (42.0%)	988	391 (39.6%)	190	56 (29.5%)	2815	118 (4.2%)	1037	310 (29.9%)	163	39 (23.9%)
**Demographics**	*N* avail.	*N* (%)	*N* avail.	*N* (%)	*N* avail.	*N* (%)	*N* avail.	*N* (%)	*N* avail.	*N* (%)	*N* avail.	*N* (%)	*N* avail.	*N* (%)	*N* avail.	*N* (%)
Age (median [IQR])	1276	71.5 [57.1, 82.6]	633	73.0 [56.0, 84.0]	411	66.0 [53.0, 79.0]	988	59.0 [46.0,75.0]	190	73.5 [59.3, 82.0]	2815	60.0 [50.0, 68.0]	1037	70.0 [57.0, 82.0]	163	60.0 [48.0–74.0]
Sex (male)	1276	742 (58.2%)	633	364 (57.5%)	411	252 (61.0%)	988	581 (58.8%)	190	120 (63.1%)	2815	1437 (51.0%)	1037	573 (55.3%)	163	95 (58.3%)
Non-White ethnicity	960	379 (39.5%)	546	55 (10.0%)	390	156 (40.0%)	817	607 (74.3%)	190	46 (24.2%)	2815	2815 (100.0%)	892	306 (34.3%)	–	–
**Comorbidities**	*N* avail.	*N* (%)	*N* avail.	*N* (%)	*N* avail.	*N* (%)	*N* avail.	*N* (%)	*N* avail.	*N* (%)	*N* avail.	*N* (%)	*N* avail.	*N* (%)	*N* avail.	*N* (%)
Hypertension	1276	695 (54.5%)	633	321 (50.7%)	411	172 (42.0%)	988	309 (31.3%)	190	117 (61.6%)	2815	821 (29.2%)	1037	637 [61.4%)	163	55 (33.7%)
Diabetes mellitus	1276	439 (34.4%)	633	163 (25.8%)	411	105 (26.0%)	988	286 (28.9%)	190	71 (37.4%)	2815	371 (13.2%)	1037	358 (34.5%)	163	27 (16.6%)
Heart failure	1276	117 (9.2%)	633	137 (21.6%)	410	–	988	52 (5.3%)	190	33 (17.4%)	2815	236 (8.4%)^2^	1037	178 (17.2%)	163	15 (9.2%)
Ischaemic heart diseases	1276	185 (14.5%)	633	152 (24.0%)	409	108 (26.0%)^1^	–	–	190	52 (27.4%)	–	–	1037	245 (23.6%)	163	21 (12.9%)
COPD	1276	141 (11.1%)	633	115 (18.2%)	409	27 (6.6%)	988	64 (6.5%)	190	41 (21.6%)	2815	17 (0.6%)	1037	152 (14.7%)	–	–
Asthma	1276	174 (13.6%)	633	112 (17.7%)	409	41 (10.0%)	988	85 (8.6%)	190	27 (14.2%)	–	–	1037	169 (16.3%)	–	–
Chronic kidney disease	1276	234 (18.3%)	633	111 (17.5%)	410	40 (9.8%)	988	110 (11.1%)	190	59 (31.1%)	2815	56 (2.0%)	1037	274 (26.4%)	163	9 (5.5%)
**Blood biomarker**	*N* avail.	Median [IQR]	*N* avail.	Median [IQR]	*N* avail.	Median [IQR]	*N* avail.	Median [IQR]	*N* avail.	Median [IQR]	*N* avail.	Median [IQR]	*N* avail.	Median [IQR]	*N* avail.	Median [IQR]
Albumin (g/L)	1153	37.0 [33.0, 40.0]	501	32.0 [29.0, 36.0]	390	38.0 [35.0, 42.0]	863	36.0 [31.0, 40.0]	190	30.0 [27.0, 33.0]	2404	38.1 [35.1, 40.5]	993	32.0 [28.0, 35.0]	120	39.0 [36.0–43.0]
C-reactive protein (CRP, mg/L)	1240	80.0 [36.0, 141.6]	545	75.0 [25.0, 150.0]	403	97.0 [45.0, 179.0]	974	76.5 [25.0, 153.8]	190	77.0 [36.3, 138.3]	2393	2.3 [0.8, 9.0]	970	89.0 [33.2, 157.0]	163	48.0 [16.0–113.0]
Urea (mmol/L)	1221	7.1 [4.6, 11.7]	563	6.95 [4.8, 10.6]	375	6.0 [4.0, 9.4]	489	7.4 [4.6, 12.5]	–	–	–	–	1018	6.8 [4.6, 11.8]	154	5.5 [4.3–7.5]
Estimated GFR	1254	65.0 [41.0, 86.0]	377	62.0 [40.0, 81.0]	407	77.0 [54.0, 96.0]	965	74.0 [49.0, 100.0]	190	68.0 [43.3. 88.0]	2433	103.1 [88.2, 117.5]	757	54.0 [29.0, 72.0]	163	84.0 [57.0–99.0]
Haemoglobin (g/L)	1223	127.0 [112.0, 141.0]	561	128.0 [111.0, 143.0]	410	130.0 [112.0, 143.0]	987	125.0 [108.0, 139.0]	190	129.0 [110.0, 141.0]	2584	124.0 [113.0, 135.0]	1009	130.0 [113.0, 144.0]	163	139.0 [129.0–148.0]
Lymphocyte count (× 10^9^/L)	1221	0.9 [0.7, 1.3]	561	1.0 [0.7, 1.4]	410	0.9 [0.6, 1.4]	987	0.9 [0.6, 1.3]	190	0.9 [0.6, 1.2]	2584	1.5 [1.1, 1.9]	1011	0.9 [0.7, 1.4]	153	1.1 [0.8–1.5]
Neutrophil count (× 10^9^/L)	1220	5.4 [3.8, 7.7]	560	5.8 [4.2, 8.8]	410	5.9 [3.9, 8.2]	986	5.0 [3.5, 8.1]	190	5.2 [3.5, 7.4]	2584	3.5 [2.7, 4.7]	1011	5.5 [3.8, 8.2]	153	4.4 [3.0–7.1]
Neutrophil/lymphocyte ratio	1218	5.6 [3.4, 9.5]	559	5.8 [3.4, 10]	410	6.0 [4.0, 10.0]	986	5.6 [3.2, 10.1]	190	5.7 [3.6, 9.8]	2584	2.3 [1.7, 3.5]	1011	5.6 [3.2, 10.2]	153	4.3 [2.4–7.7]
Lymphocyte/CRP ratio	1196	1.2 [0.6, 3.2]	559	1.3 [0.5, 4.6]	402	1.0 [0.4, 2.4]		0.0 [0.0, 0.0]	190	1.1 [0.5, 2.7]	2362	0.7 [0.1, 2.0]	962	1.1 [0.5, 3.3]	–	–
Platelet count (× 10^9^/L)	1224	213.0 [161.8, 274.0]	560	231 [176.8, 303.5]	409	221.0 [169.0, 280.0]	986	209.0 [161.0, 275.8]	190	207.5 [150.3, 268.5]	2584	223.0 [179.8, 273.0]	1008	218.0 [165.0, 287.2]	163	205.0 [160.0–279.0]
**Physiological parameters**	*N* avail.	Median [IQR]	*N* avail.	Median [IQR]	*N* avail.	Median [IQR]	*N* avail.	Median [IQR]	*N* avail.	Median [IQR]	*N* avail.	Median [IQR]	*N* avail.	Median [IQR]	*N* avail.	Median [IQR]
NEWS2 Total Score	1262	2.0 [1.0, 4.0]	529	3.0 [2.0, 5.0]	404	5.0 [3.0, 7.0]	744	3.0 [1.0, 5.0]	190	3.0 [2.0, 5.0]	2804	1.0 [0.0, 3.0]	1019	4.0 [2.0, 7.0]	163	5.0 [3.0–7.0]
Heart rate	1273	85.0 [75.0, 94.0]	560	90.5 [82.0, 102.0]	410	94.0 [81.0, 107.0]	752	85.0 [75.0, 95.0]	190	82.0 [71.0, 95.0]	2812	81.0 [76.9, 85.8]	1028	90.0 [79.0, 104.0]	160	89.5 [75.3–100.8]
Oxygen saturation	1273	96.0 [95.0, 98.0]	561	97.0 [96.0, 99.0]	410	96.0 [94.0, 98.0]	712	96.0 [95.0, 97.0]	190	95.0 [94.0, 96.0]	2797	97.8 [97.0, 98.2]	1029	96.0 [94.0, 98.0]	163	95.0 [92.0–97.0]
Oxygen flow rate (L/min)	1271	0.0 [0.0, 4.0]	260	3.0 [2.0, 8.0]	403	2.0 [0.0, 10.0]	978	0.0 [0.0, 0.0]	190	2.0 [0.0, 3.0]	–	–	1017	0.0 [0.0, 4.0]	125	1.0 [0.0–2.0]
Respiration rate	1273	19.0 [18.0, 21.0]	561	20.0 [19.0, 24.0]	410	24.0 [20.0, 28.0]	755	19.0 [18.0, 22.0]	190	20.0 [18.0, 21.0]	2811	20.0 [19.0, 21.0]	1020	20.0 [18.0, 25.0]	160	24.0 [20.0–28.0]
Systolic blood pressure	1273	125.0 [112.0, 139.0]	555	137.0 [123.0, 152.0]	411	131.0 [115.0, 143.0]	751	125.0 [115.0, 140.0]	190	123.0 [111.0, 140.8]	1431	120.0 [110.0, 128.0]	1022	128.0 [113.0, 144.0]	160	129 [116.0–142.8]
Diastolic blood pressure	1273	71.0 [62.0, 80.0]	555	78.0 [70.0, 85.0]	411	73.0 [64.0, 81.0]	751	74.0 [66.0, 81.0]	190	72.0 [64.3, 82.0]	1433	71.0 [65.0, 78.0]	1022	75.0 [67.0, 84.0]	160	77.0 [69.0–87.0]
Temperature	1273	36.9 [36.6, 37.4]	558	36.9 [36.7, 37.5]	410	37.3 [36.8, 38.1]	750	36.9 [36.4, 37.5]	190	37.2 [36.7, 37.9]	2815	36.5 [36.3, 36.7]	1029	36.8 [36.2, 37.5]	162	37.2 [36.5–38.3]

^1^Measured as ‘cardiovascular disease’ at UCH because separate measures of ‘heart failure’ and ‘ischaemic heart diseases’ were unavailable

^2^Measured as overall ‘heart disease’ in the Wuhan cohort

Logistic regression models were used to assess independent associations between each variable and severe COVID-19 outcome (ICU transfer/death) in the KCH cohort. Additional file 3: Table S3 presents odds ratios adjusted for age and sex (model 1) and comorbidities (model 2), sorted by effect size. Increased odds of transfer to the ICU or death by 14 days were associated with NEWS2 score, oxygen flow rate, respiratory rate, CRP, neutrophil count, urea, neutrophil/lymphocyte ratio, heart rate, and temperature. Reduced odds of severe outcomes were associated with lymphocyte/CRP ratio, oxygen saturation, estimated GFR, and albumin.

### Evaluating NEWS2 score for prediction of severe COVID-19 outcome

**Fig. 1:**
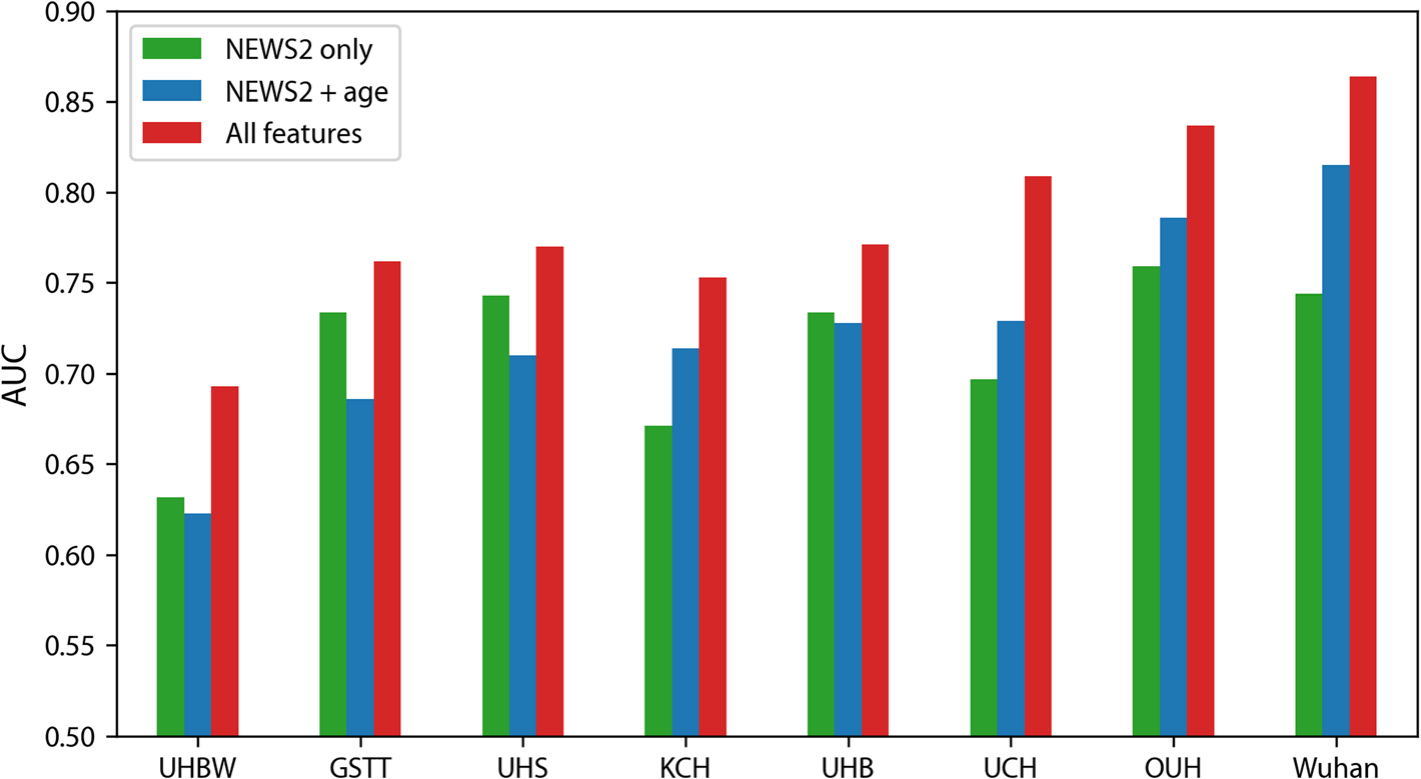
Improvement in the area under the curve (AUC) for supplemented NEWS2 model for 14-day ICU/death at training and validation sites

**Fig. 2:**
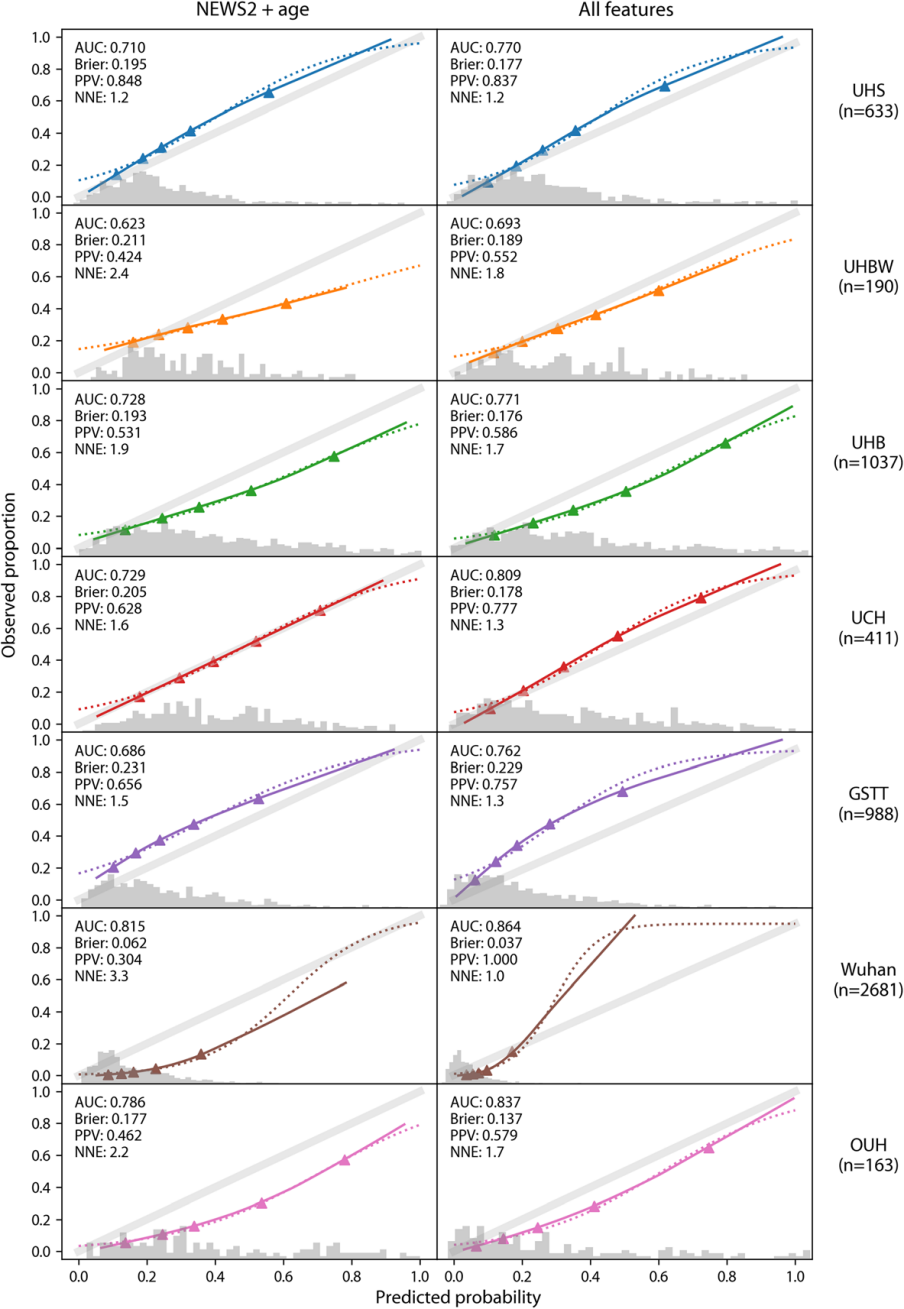
Calibration (logistic and LOESS curves) of supplemented NEWS2 model for 14-day ICU/death model at validation sites

Logistic regression models were used to evaluate a baseline model containing hospital admission NEWS2 score and age for the prediction of severe COVID-19 outcomes at 14 days. Internally validated discrimination for the KCH training sample was moderate (AUC = 0.700; 95% confidence interval (CI) 0.680, 0.722; Brier score = 0.192; 0.186, 0.197; Table 2). Discrimination remained poor-to-moderate in UK validation sites (AUC = 0.623 to 0.729) but was moderate-to-good in Norway (AUC = 0.786) and Wuhan hospitals (AUC = 0.815) (Figs. 1 and 2). Calibration was inconsistent with risks underestimated in some sites (UHS, GSTT) and overestimated in others (UHBW, UHB; Fig. 2).

**Table 2:** KCH internally validated predictive performance (*n* = 1276) based on nested repeated cross-validation

		NEWS2 + age, mean (95% CI)	All features, mean (95% CI)
14-day ICU/death	AUC	0.700 [0.680, 0.722]	0.735 [0.715, 0.757]
	Brier score	0.192 [0.186, 0.197]	0.183 [0.177, 0.189]
	Sensitivity^1^	0.778 [0.747, 0.815]	0.735 [0.702, 0.772]
	Specificity^1^	0.478 [0.445, 0.509]	0.592 [0.562, 0.621]

^1^Calculated at 30% probability threshold. AUC based on repeated, nested cross-validation (inner loop, 10-folds; outer loop = 10-folds/1000 repeats). Missing values imputed at each outer loop with *k*-nearest neighbour (kNN) imputation

### Supplementing NEWS2 with routinely collected blood and physiological parameters

We considered whether routine blood and physiological parameters could improve risk stratification for medium-term COVID-19 outcome (ICU transfer/death at 14 days). When adding demographic, blood, and physiological parameters to NEWS2, nine features were retained following LASSO regularisation, in order of effect size: NEWS2 score, supplemental oxygen flow rate, urea, age, oxygen saturation, CRP, estimated GFR, neutrophil count, and neutrophil/lymphocyte ratio. Notably, comorbid conditions were not retained when added in subsequent models, suggesting most of the variance explained was already captured by the included parameters. Internally validated discrimination in the KCH training sample was moderate (AUC = 0.735; 95% CI 0.715, 0.757) but improved compared to ‘NEWS2 + age’ (Table 2). This improvement over NEWS2 alone was replicated in validation samples (Fig. 1). The supplemented model continued to show evidence of substantial miscalibration.

#### Sensitivity analyses

For the 3-day endpoint, 13% of patients at KCH (*n* = 163) and between 16 and 29% of patients in the UK and Norway were transferred to the ICU or died (Table 1). The 3-day model retained just two parameters following regularisation: NEWS2 score and supplemental oxygen flow rate. For the baseline model (‘NEWS2 + age’), discrimination was moderate at internal validation (AUC = 0.764; 95% CI 0.737, 0.794; Additional file 4: Table S4) and external validation (AUC = 0.673 to 0.755), but calibration remained poor (Additional file 5: Figure S1). Moreover, the supplemented model (‘NEWS2 + oxygen flow rate’) showed smaller improvements in discrimination compared to those seen at 14 days. For the KCH training cohort, internally validated AUC increased by 0.025: from 0.764 (95% CI 0.737, 0.794) for ‘NEWS2 + age’ to 0.789 (0.763, 0.819) for the supplemented model (‘NEWS2 + oxygen flow rate’). At external validation, improvements were modest (UHBW, OUH) or negative (GSTT) in some sites, but more substantial in others (UHS, UCH). Moreover, model calibration was considerably worse for the supplemented 3-day model (Additional file 5: Figure S1).

We found no evidence of difference by sex (results not shown) and the findings were consistent when additionally adjusting for ethnicity in the subset of individuals with ethnicity data and when excluding nosocomial patients (Additional file 6: Table S5). Discrimination for the alternative baseline model of ‘NEWS2 only’ (Additional file 7: Table S6) showed a similar pattern of results as those for ‘NEWS2 + age’, except that improvements in discrimination for the supplemented model (‘All features’) were larger in most sites.

#### Decision curve analysis

**Fig. 3:**
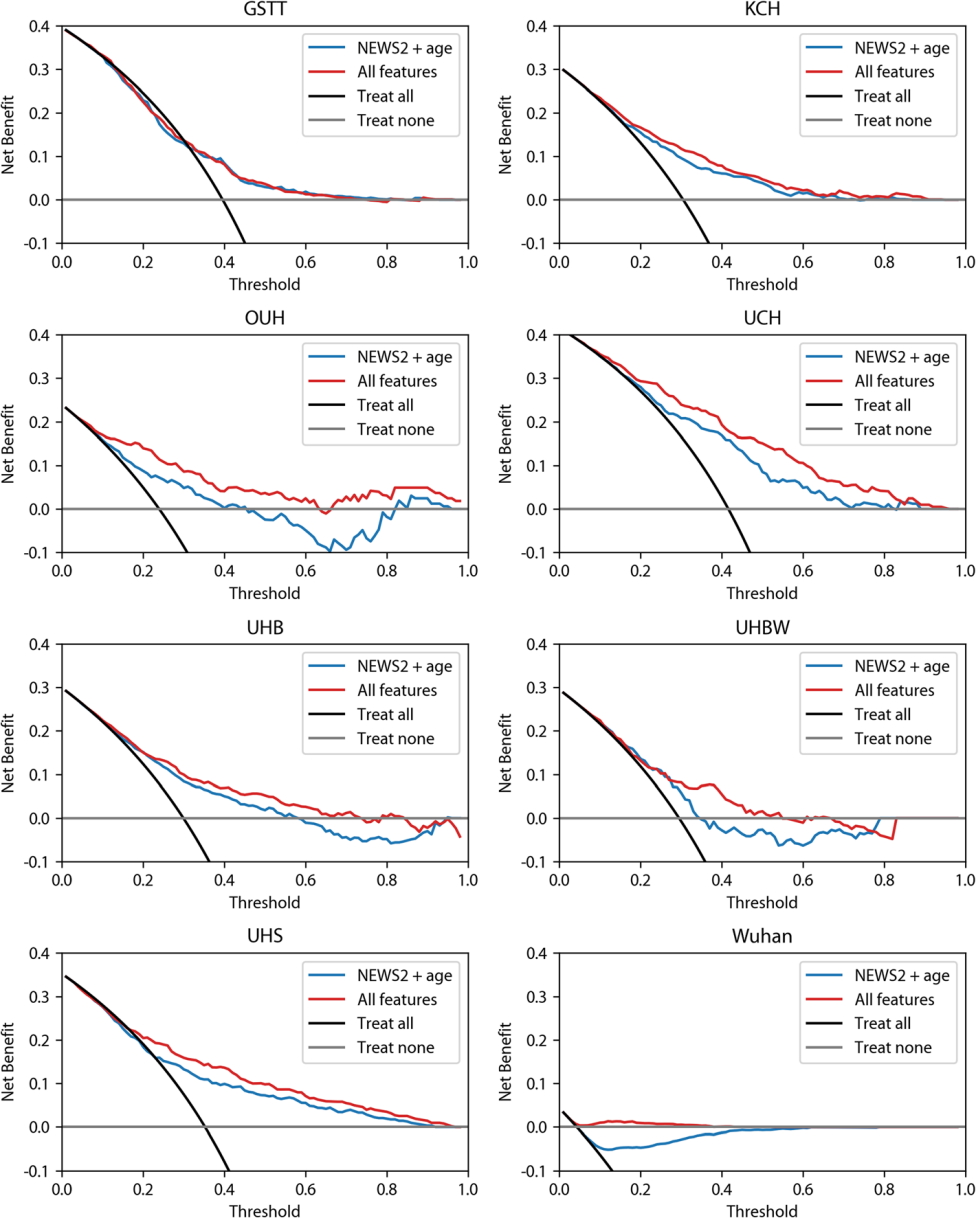
Net benefit of supplemented NEWS2 model for 14-day ICU/death compared to default strategies (‘treat all’ and ‘treat none’) at training and validation sites

Decision curve analysis for the 14-day endpoint is presented in Fig. 3. At KCH, the baseline model (‘NEWS2 + age’) offered small increments in net benefit compared to the ‘treat all’ and ‘treat none’ strategies for risk thresholds in the range 25 to 60%. This was replicated in all validation cohorts except for UHBW and OUH where the net benefit for ‘NEWS2 + age’ was lower than the ‘treat none’ strategy beyond the 40% risk threshold. The supplemented model (‘All features’) improved upon ‘NEWS2 + age’ and the two default strategies in most sites across the range 20 to 80%, except for (i) UHBW, where ‘treat none’ was superior beyond thresholds of 55%, and (ii) GSTT, where ‘treat all’ was superior up to a threshold of 30% and no improvement was seen for the supplemented model.

For the 3-day endpoint, the improvement in net benefit for the supplemented model over the two default strategies was smaller, compared to the improvements seen at 14 days (Additional file 8: Figure S2). At three sites (UHBW, GSST, and Wuhan), neither the baseline (‘NEWS2 + age’) nor the supplemented (‘All features’) models offered any improvement over the ‘treat all’ or ‘treat none’ strategies. At KCH and UHS, net benefit for ‘NEWS2 + age’ was higher than the default strategies for a range of risk thresholds but was not increased further by the supplemented (‘NEWS2 + oxygen flow rate’) model.

## Discussion

### Principal findings

This study is amongst the first to systematically evaluate NEWS2 for severe COVID-19 outcome and carry out external validation at multiple international sites (five UK NHS Trusts, one hospital in Norway, and two hospitals in Wuhan, China). We found that while ‘NEWS2 + age’ had moderate discrimination for short-term COVID-19 outcome (3-day ICU transfer/death), it showed poor-to-moderate discrimination for the medium-term outcome (14-day ICU transfer/death). Thus, while NEWS2 may be effective for short-term (e.g. 24 h) prognostication, our results question its suitability as a screening tool for medium-term COVID-19 outcome. Risk stratification was improved by adding routinely collected blood and physiological parameters, and discrimination in supplemented models was moderate-to-good. However, the model showed evidence of miscalibration, with a tendency to underestimate risks in external sites. The derived model for 14-day ICU transfer/death included nine parameters: NEWS2 score, supplemental oxygen flow rate, urea, age, oxygen saturation, CRP, estimated GFR, neutrophil count, and neutrophil/lymphocyte ratio. Notably, pre-existing comorbidities did not improve risk prediction and were not retained in the final model. This was unexpected but may indicate that the effect of pre-existing health conditions could be manifest through some of the included blood or physiological markers.

Overall, this study overcomes many of the factors associated with a high risk of bias in the development of prognostic models for COVID-19 [13] and provides some evidence to support the supplementation of NEWS2 for clinical decisions with these patients.

### Comparison with other studies

A systematic review of 10 prediction models for mortality in COVID-19 infection [10] found broad similarities with the features retained in our models, particularly regarding CRP and neutrophil levels. However, existing prediction models suffer several methodological weaknesses including overfitting, selection bias, and reliance on cross-sectional data without accounting for censoring. Additionally, many existing studies have relied on single-centre or ethnically homogenous Chinese cohorts, whereas the present study shows validation across multiple and diverse populations. A key strength of our study is the robust and repeated external validation across national and international sites; however, evidence of miscalibration suggests we should be cautious when attempting to generalise these findings. Future research should include larger collaborations and aim to develop ‘from onset’ population predictions.

NEWS2 is a summary score derived from six physiological parameters, including oxygen supplementation. Lack of evidence for NEWS2 use in COVID-19 especially in primary care has been highlighted [9]. The oxygen saturation component of physiological measurements added value beyond NEWS2 total score and was retained following regularisation for 14-day endpoints. This suggests some residual association over and above what is captured by the NEWS2 score and reinforces Royal College of Physicians guidance that the NEWS2 score ceilings with respect to respiratory function [42].

Cardiac disease and myocardial injury have been described in severe COVID-19 cases in China [2, 23]. In our model, blood Troponin-T, a marker of myocardial injury, had additional salient signal but was only measured in a subset of our cohort at admission, so it was excluded from our final model. This could be explored further in larger datasets.

### Strengths and limitations

Our study provides a risk stratification model for which we obtained generalisable and robust results across seven national and international sites with differing geographical catchment and population characteristics. It is amongst the first to evaluate NEWS2 at hospital admission for severe COVID-19 outcome and amongst a handful to externally validate a supplemented model across multiple sites.

However, some limitations must be acknowledged. First, there are likely to be other parameters not measured in this study that could substantially improve the risk stratification model (e.g. radiological features, obesity, or comorbidity load). These parameters could be explored in future work but were not considered in the present study to avoid limiting the real-world implementation of the risk stratification model. Second, our models showed better performance in UK secondary care settings amongst populations with higher rates of severe COVID-19 disease. Therefore, further research is needed to investigate the suitability of our model for primary care settings which have a high prevalence of mild disease severities and in community settings. This would allow us to capture variability at earlier stages of the disease and trends in patients not requiring hospital admission. Third, while external validation across multiple national and international sites represents a key strength, we did not have access to individual participant data and model development was limited to a single site (KCH). Although we benefited from existing infrastructure to support rapid data analysis, we urgently need infrastructure to support data sharing between sites to address some of the limitations of the present study (e.g. miscalibration) and improve the transferability of these models. Not only would this facilitate external validation, but more importantly, it would allow multi-site prediction models to be developed using pooled, individual participant data [43]. Fourth, our analyses would have excluded patients who experienced severe COVID-19 outcome at home or at another hospital, after being discharged from a participating hospital. Fifth, our model was restricted to blood and physiological parameters measured at hospital admission. This was by design and reflected the aim of developing a screening tool for risk stratification at hospital admission. However, future studies should explore the extent to which risk stratification could be improved by incorporating repeated measures of NEWS2 and relevant biomarkers.

## Conclusions

The NEWS2 early warning score is in near-universal use in UK NHS Trusts since March 2019 [15], but little is known about its use for COVID-19 patients. Here, we showed that NEWS2 and age at hospital admission had poor-to-moderate discrimination for medium-term (14-day) severe COVID-19 outcome, questioning its use as a tool to guide hospital admission. Moreover, we showed that NEWS2 discrimination could be improved by adding eight blood and physiological parameters (supplemental oxygen flow rate, urea, age, oxygen saturation, CRP, estimated GFR, neutrophil count, neutrophil/lymphocyte ratio) that are routinely collected and readily available in healthcare services. Thus, this type of model could be easily implemented in clinical practice, and predicted risk score probabilities of individual patients are easy to communicate. At the same time, although we provided some evidence of improved discrimination vs. NEWS2 and age alone, given miscalibration in external sites, our proposed model should be used as a complement and not as a replacement for clinical judgement.

## Supplementary Information

**Additional file 1: Table S1.** SNOMED terms.

**Additional file 2: Table S2.** F1, precision and recall for NLP comorbidity detection.

**Additional file 3: Table S3.** Logistic regression models for each blood and physiological measure tested separately in the KCH training cohort, for 14- and 3-day ICU/death.

**Additional file 4: Table S4.** Internally validated discrimination for KCH training sample based on nested repeated cross-validation.

**Additional file 5: Figure S1.** Calibration (logistic and LOESS curves) of supplemented NEWS2 model for 3-day ICU/death model at validation sites.

**Additional file 6: Table S5.** Univariate logistic regression models for sensitivity analyses showing odds ratios of ICU/death at 3- and 14-days for subsets of the training cohort.

**Additional file 7: Table S6.** Discrimination for all models in training and validation cohorts, including alternative baseline model of ‘NEWS2 only’.

**Additional file 8: Figure S2.** Net benefit of supplemented NEWS2 model for 3-day ICU/death compared to default strategies (‘treat all’ and ‘treat none’) at training and validation sites.

## Data Availability

Code and pre-trained models are available at https://github.com/ewancarr/NEWS2-COVID-19 and openly shared for testing in other COVID-19 datasets. Source text from patient records used at all sites in the study will not be available due to inability to safely fully anonymise up to the Information Commissioner Office (ICO) standards and would be likely to contain strong identifiers (e.g. names, postcodes) and highly sensitive data (e.g. diagnoses). A subset of the KCH dataset limited to anonymisable information (e.g. only SNOMED codes and aggregated demographics) is available on request to researchers with suitable training in information governance and human confidentiality protocols subject to approval by the King’s College Hospital Information Governance committee; applications for research access should be sent to kch-tr.cogstackrequests@nhs.net. This dataset cannot be released publicly due to the risk of re-identification of such granular individual-level data, as determined by the King’s College Hospital Caldicott Guardian. The GSTT dataset cannot be released publicly due to the risk of re-identification of such granular individual-level data, as determined by the Guy’s and St Thomas’s Trust Caldicott Guardian. The UHS dataset cannot be released publicly due to the risk of re-identification of such granular individual-level data, as determined by the University Hospital Southampton Caldicott Guardian. The UCH data cannot be released publicly due to conditions of regulatory approvals that preclude open access data sharing to minimise the risk of patient identification through granular individual health record data. The authors will consider specific requests for data sharing as part of academic collaborations subject to ethical approval and data transfer agreements in accordance with the GDPR regulations. The Wuhan dataset used in the study will not be available due to the inability to fully anonymise in line with ethical requirements. Applications for research access should be sent to TS and details will be made available via https://covid.datahelps.life/prediction/. The OUH dataset cannot be released publicly due to the risk of re-identification of such granular individual-level data.

## Footnotes

1 https://github.com/ewancarr/NEWS2-COVID-19
