# Supplementary figures and images for: Evaluation and improvement of the National Early Warning Score (NEWS2) for COVID-19: a multi-hospital study
Source: BMC Med. 2021 Jan 21;19:23. doi: 10.1186/s12916-020-01893-3 (PMC7817348; doi:10.1186/s12916-020-01893-3)

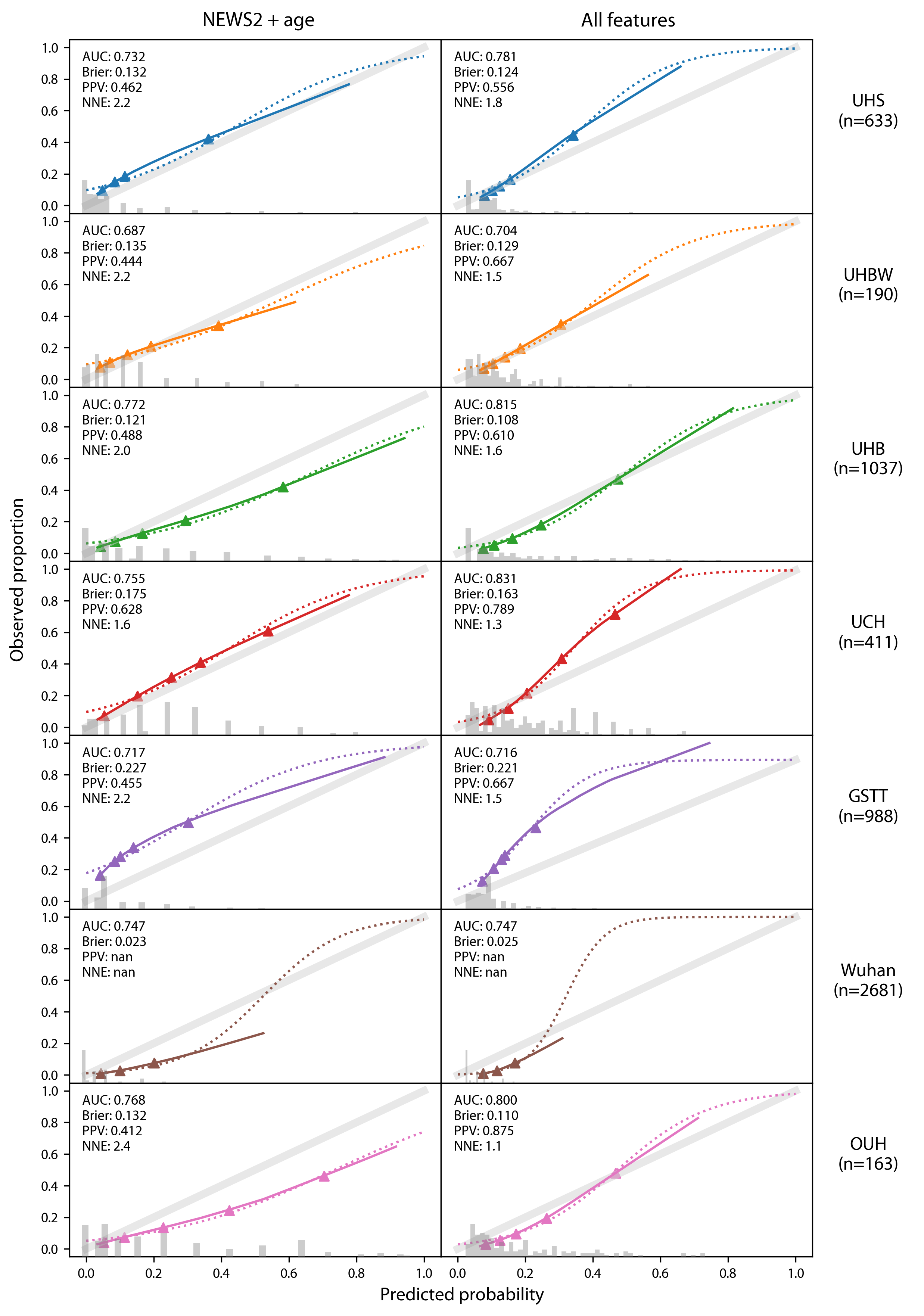

Supplement: Supplementary file 5 — Additional file 5: Figure S1. Calibration (logistic and LOESS curves) of supplemented NEWS2 model for 3-day ICU/death model at validation sites. [file 12916_2020_1893_MOESM5_ESM.png]

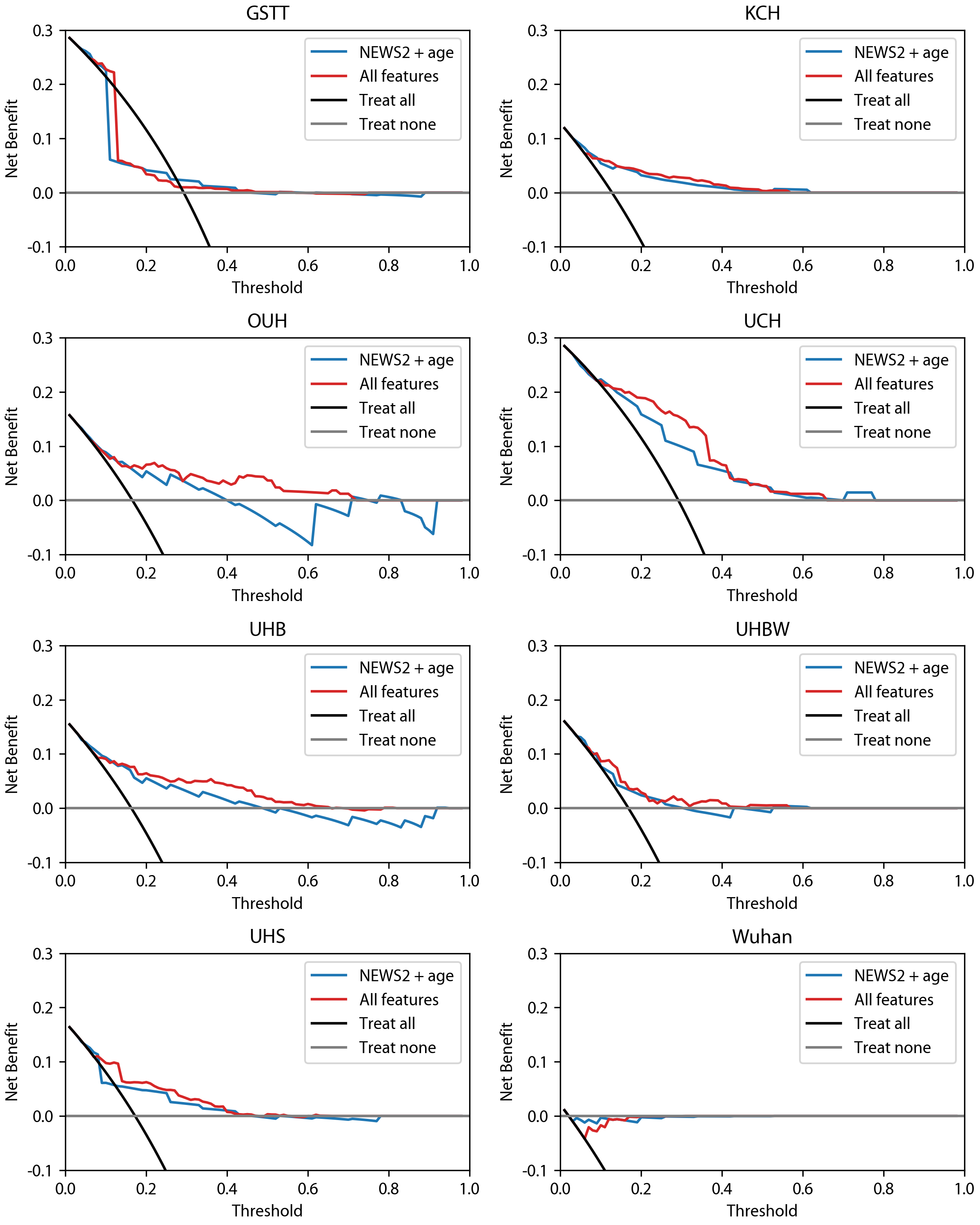

Supplement: Supplementary file 8 — Additional file 8: Figure S2. Net benefit of supplemented NEWS2 model for 3-day ICU/death compared to default strategies (‘treat all’ and ‘treat none’) at training and validation sites. [file 12916_2020_1893_MOESM8_ESM.png]
